# Antioxidant, antihyperglycemic, and antihyperlipidemic properties of *Chimonanthus salicifolius* S. Y. Hu leaves in experimental animals: modulation of thioredoxin and glutathione systems, renal water reabsorption, and gut microbiota

**DOI:** 10.3389/fnut.2023.1168049

**Published:** 2023-04-28

**Authors:** Ruixia Dong, Junjie Pan, Guangshan Zhao, Qiuyan Zhao, Shiqiong Wang, Ning Li, Lianjun Song, Xianqing Huang, Shuxing Miao, Junhui Ying, Fangying Wu, Dongxu Wang, Kejun Cheng, Daniel Granato, Qiuyan Ban

**Affiliations:** ^1^College of Horticulture, Jinling Institute of Technology, Nanjing, China; ^2^College of Forestry Science and Technology, Lishui Vocational and Technical College, Lishui, China; ^3^State Key Laboratory of Tea Plant Biology and Utilization, School of Tea and Food Science and Technology, Anhui Agricultural University, Hefei, China; ^4^Chemical Biology Center, Lishui Institute of Agriculture and Forestry Sciences, Lishui, China; ^5^Innovation Team of Food Nutrition and Safety Control, College of Food Science and Technology, Henan Agricultural University, Zhengzhou, China; ^6^School of Grain Science and Technology, Jiangsu University of Science and Technology, Zhenjiang, China; ^7^Bioactivity and Applications Lab, Department of Biological Sciences, Faculty of Science and Engineering, University of Limerick, Limerick, Ireland; ^8^Department of Tea Science, College of Horticulture, Henan Agricultural University, Zhengzhou, China

**Keywords:** *Chimonanthus salicifolius* S. Y. Hu leaves, antioxidant, metabolic syndrome, renal water reabsorption, gut microbiota

## Abstract

**Introduction:**

Excessive calorie intake and physical inactivity have dramatically increased nutrient overload-associated disease, becoming a global public health issue. *Chimonanthus salicifolius* S. Y. Hu (*CHI*) is a homology plant of food and medicine in China and shows several health benefits.

**Methods:**

This work investigated the antioxidant activity, the alleviating effects, and the mechanism of action on diabetes and hyperlipidemia of *CHI* leaves.

**Results and discussion:**

Results showed that *CHI* leaves infusion displayed *in vitro* antioxidant activity measured by ABTS and ferric reducing antioxidant power methods. In wild-type Kunming mice, *CHI* leaves infusion consumption activated the hepatic antioxidant enzymes, including glutathione reductase, glutathione *S*-transferase, glutathione peroxidase and thioredoxin reductase as well as thioredoxin reductase 1. In alloxan-induced type 1 diabetic mice, *CHI* leaves infusion ameliorated diabetic symptoms, including polyuria, polydipsia, polyphagia and hyperglycemia, in a dose-dependent and time-course manners. The mechanism involved *CHI* leaves up-regulating renal water reabsorption associated protein – urine transporter A1–and promoting the trafficking of urine transporter A1 and aquaporin 2 to the apical plasma membrane. Despite this, in high-fat diet-induced hyperlipidemic golden hamsters, *CHI* leaves powder did not significantly effect on hyperlipidemia and body weight gain. This might be attributed to *CHI* leaves powder increasing the calorie intake. Interestingly, we found that *CHI* leaves extract containing a lower dose of total flavonoid than *CHI* leaves powder pronouncedly reduced the levels of total cholesterol, triglyceride, and low-density lipoprotein cholesterol in serum in golden hamsters fed a high-fat diet. Furthermore, *CHI* leaves extract elevated the diversity of gut microbiota and the abundance of *Bifidobacterium* and *Ruminococcaceae_UCG-014.* It also decreased the abundance of *Lactobacillus* at the genus level in golden hamsters fed a high-fat diet. Overall, *CHI* leaves benefit oxidative stress prevention and metabolic syndrome amelioration *in vivo.*

## Graphical Abstract

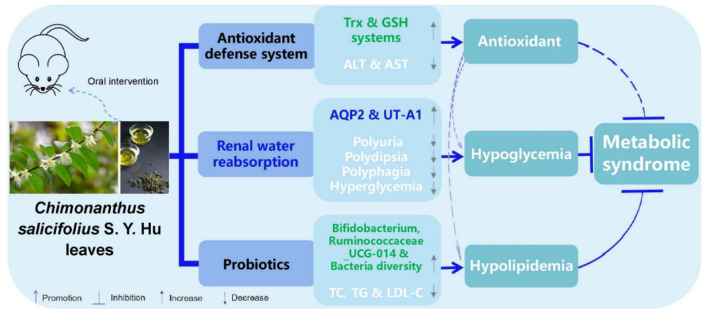

## Introduction

The overnutrition-associated diseases, including systemic oxidative stress response, obesity, non-alcoholic fatty liver, and type 2 diabetes mellitus induced by westernized diet patterns and sedentary lifestyle, have become a global public health issue (1–4). Redox homeostasis contributes to the alleviation of metabolic disturbances (5, 6). Long-term low-level oxidative stress induces inflammatory response and aggravates metabolic syndrome. Besides, the metabolism toxicities and secondary metabolites of glucose and lipid strongly enhance oxidative stress, trigger oxidative modification of biomolecules, and induce alterations in redox metabolic regulation (5, 6). To equilibrate the oxidative stress and maintain cellular redox homeostasis, the antioxidant defense systems, including the thioredoxin (Trx) system comprising of Trx and thioredoxin reductase (TrxR), the glutathione (GSH) system comprising of GSH and glutathione reductase (GR) coupled with glutaredoxin and the nuclear factor-E2-related factor 2 (Nrf2) pathway intricately interact with each other (7). In addition, the relationship is intricate between antioxidant defense systems and glycolipid metabolism (5).

The liver, pancreas, adipose tissue, and skeletal muscle are affected by metabolic diseases and energy homeostasis. Increasing evidence shows kidney participates and plays a crucial role in maintaining glucose homeostasis. Renal-specific sodium-glucose cotransporter 2 (SGLT2) in proximal tubules performs approximately 90% of active renal glucose reabsorption; SGLT2 was considered a therapeutic target for anti-hyperglycemia by promoting glycosuria, which was considered a novel therapeutic strategy for type 2 diabetes (8–10). Though the approach aiming at enhancing glycosuria by inhibiting renal-specific SGLT2 has been validated effectively in diabetic patients, SGLT2 inhibitors increase compensatory polyphagia and excessive water loss, and elevate hematocrit and the incidence of urinary tract infection (8, 11, 12). In addition, gut microbiota was another crucial organ involved in maintaining the host’s health via interactions with the liver and brain. Intestinal flora and its metabolites play an important role in regulating energy homeostasis and host’s health (3, 13–16). Intestinal flora is affected by calories intake, and nutrient overload could cause gut microbiota dysbiosis, alter the metabolites of intestinal flora and enhance the systemic oxidative stress level and inflammatory response (3, 13, 14).

“Shi-Liang-tea” is widely consumed as a functional beverage with a protective effect on the gastrointestinal track among some ethnic minorities, especially She-minority in China. Its basal resource, *Chimonanthus salicifolius* S.Y. Hu (*CHI*), a Chinese endemic plant, belongs to the *Calycanthaceae* family and the *Chimonanthus Lindley* genus, was approved as a new food raw material by the National Health Commission of China in 2014. “Shi-Liang-tea” also serves as a folk medicine due to its significant effect on eliminating food (17), strengthening the spleen, and stopping diarrhea. It was included in concocts standard of traditional Chinese medicine in Zhejiang Province in 2015. As the homology of food and medicine, *CHI* leaves contain various chemical ingredients, including volatile oil, flavonoids, and alkaloid (17, 18). Furthermore, *CHI* leaves show numerous biological activities, including antioxidant, anti-inflammatory, anti-hyperlipidemia, anti-hypertensive, and anti-microbial effects (19–22). Previous studies showed that the water extraction of *CHI* leaves alleviated the lipid dysmetabolism in mice with acute hyperlipidemia caused by intraperitoneal injection of egg yolk and rats with hyperlipidemia caused by high-fat diets as reflected by the decreased levels of cholesterol (TC), triglyceride (TG) and low-density lipoprotein cholesterol (LDL-C) in serum (23). However, no report related the regulating effects of *CHI* leaves on lipid metabolism to modifying gut microbiota. *Chimonanthus nitens* Oliv., pertains to *Chimonanthus* Lindl, has similar ingredients to *CHI* (24). *Chimonanthus nitens* Oliv. was reported to show hypoglycemic activity, as evidenced by the significant inhibitory effect on α-glucosidase activity *in vitro* (25) and anti-hyperlipidemic activity, as reflected by lowered levels of TC, TG, and LDL-C in serum of diabetic mice post *Chimonanthus nitens* Oliv. treatment (26). These reports mentioned above suggest that *CHI* leaves may have the biological activity to modify intestinal flora and regulate glycolipid metabolism.

Diet represents the most important modifiable factor in preventing disease, and plant-based dietary patterns are associated with a lower risk of metabolic syndrome and its associated disease. It is an economical and effective strategy to alleviate the global public health issue caused by overnutrition by dietary supplements with natural products or their active ingredients. “Shi-Liang-tea” (*CHI*) beverage is considered a healthy and safe drink with several health benefits. The present study investigated the antioxidant activity, anti-hyperglycemic and anti-hyperlipidemic effects of *CHI* leaves and further explored the underlying mechanism of action on diabetes and hyperlipidemia.

## Materials and methods

### Chemical reagents and standards

*Chimonanthus salicifolius* S. Y. Hu was obtained from the Lishui Institute of Agriculture and Forestry Sciences (Lishui, China). Rutinum, isoquercitrin, kaempferol 3-rutinoside, astragalin and ALX were purchased from Sigma (St. Louis, MO, USA). Epigallocatechin (EGC), catechin (C), epicatechin (EC), (-)-epigallocatechin-3-gallate (EGCG), and (-)-epicatechin-3-gallate (ECG) (purity > 99%) for animal treatment were obtained from Ebeikar Tea & Extracts Co., Ltd. (Hangzhou, China). Glutathione reductase (from *Escherichia coli*), GSH, nicotinamide adenine dinucleotide phosphate (NADPH), bovine serum albumin (BSA), 1-chloro,-2, 4-dinitrobenzene (CDNB) and 5,5’-dithiobis (2-ni-trobenzoic acid) (DTNB) were all obtained from Sigma (St. Louis, MO, USA). Total antioxidant capacity assay kits with ABTS and FRAP methods were purchased from Nanjing Jiancheng Biotechnology Co., Ltd. (Shanghai, China). Kits for serum alanine aminotransferase (ALT), aspartate aminotransferase (AST), alkaline phosphatase (AKP), TC, TG and LDL-C as well as high-density lipoprotein cholesterol (HDL-C) were all obtained from Nanjing Jiancheng Biotechnology Co., Ltd. (Shanghai, China). The membrane of ECL Plus reagent and polyvinylidene fluoride (PVDF) were purchased from Shanghai Bio-Rad Laboratories, Inc. (Shanghai, China). Phenylmethanesulfonyl fluoride (PMSF) was obtained from Sigma. Radio-Immune Precipitation Assay (RIPA) regent and BCA protein assay kit were products of Beyotime Biotechnology (Shanghai, China). The primary antibodies against aquaporin 2 (AQP2) and the secondary antibody anti-rabbit IgG were obtained from Cell Signaling Technology Inc. (Boston, MA, USA). The primary antibody against TrxR1 was obtained from Santa Cruz (Dallas, TX, USA). The primary antibodies against urea transporter-A1 (UT-A1) and β-actin and the secondary antibody anti-mouse IgG were bought from Sigma (St. Louis, MO, USA). Other chemicals were of the highest grade available.

### *CHI* samples preparation

Standardized *CHI* infusion (*CHII*, 1/10, w/v) was prepared by immersing 1 g of dried CHI leaves in 10 mL hot water (100°C) for 10 min. The *CHI* infusion was cooled to room temperature (RT) in a water bath, filtrated by absorbent cotton, and stored frozen at −80°C. The *CHII* used for animal treatments (1/20 or 1/40, w/v) were prepared by diluting the concentrated infusion (1/10, w/v) with distilled water before use.

The dried *CHI* leaves were pre-crushed to a size of less than 12 mesh and then powdered using a bead mill with a millstone (CJM-SY-A, Terada Seisakusho Co., Ltd.) for 20 h (rotational speed of 50 to 55 rpm) to produce the matcha refer to as *CHI* leaves powder (*CHIP*) (particle size of fewer than 18 μm). The indoor temperature was below 20°C, and the relative humidity was below 50%.

The *CHI* infusion (1/10, w/v) was prepared by immersing 1 g of dried *CHI* leaves in 10 mL hot water (100°C) for 10 min and then was filtrated by absorbent cotton. The obtained suspension was centrifuged at 3,000 *g* for 20 min, and a rotary evaporator (Vacuum 0.1 MPa, 70) was used to concentrate the supernatant. The concentrates were further dried (inlet temperature 135°C, outlet temperature 85°C, feed speed 11 mL/min) by a spray mini-dryer (BUCHI MINI SPRAY DRYER B-290, BUCHI Labortechnik AG, Switzerland) refer to as *CHI* leaves extract (*CHIE*) and stored frozen at −80°C.

### HPLC assay

To measure flavonoids of *CHI*, an Agilent 1260 HPLC system equipped with a degasser, a quaternary pump, a light-tight autosampler unit set, a thermostated column compartment, and a 2,489 UV/Vis detector (360 nm) was employed (Agilent Technologies, Santa Clara, CA, USA). Chromatographic separation was achieved by an Agilent Zorbax SB-C18 column (250 mm × 4.60 mm, 5 μm) at 30°C. The mobile phase consisted of (A) 0.1% aqueous phosphoric acid and (B) acetonitrile. The gradient of solvent A was as follows: 0 min at 82% A, a linear gradient to 70% A for 10 min, then a linear gradient to 55% A for 14 min, and held for 6 min to balance the system. The injection volume was 10 μL, and the elution rate was 1 mL/min. Peaks were identified by comparison of retention times with those of standards. The major constituents of *CHI* leaves infusions are shown in Tables 1, 2.

**TABLE 1:** Flavonoids content in *CHI* leaves preparations^a^.

*CHI* leaves preparations	Flavonoids						Total flavonoids
	**Rutinum**	**Isoquercitrin**	**Kaempferol-3-O-rutinoside**	**Astragalin**	**Quercetin**	**Kaempferol**	
*CHII* (1/10, m/v; mg/mL)	0.28 ± 0.01	0.12 ± 0.00	0.72 ± 0.03	0.00 ± 0.00	0.00 ± 0.00	0.00 ± 0.00	1.13 ± 0.05
*CHIP* (mg/g)	11.11 ± 0.05	4.76 ± 0.02	28.76 ± 0.12	0.30 ± 0.00	0.06 ± 0.00	0.32 ± 0.02	45.23 ± 0.18
*CHIE* (mg/g)	4.97 ± 0.05	1.98 ± 0.05	12.77 ± 0.11	1.33 ± 0.00	0.20 ± 0.00	0.19 ± 0.01	21.59 ± 0.10

^a^Total flavonoids represent the sum of rutinum, isoquercitrin, kaempferol-3-O-rutinoside, astragalin, quercetin, and kaempferol. Flavonoids were measured with HPLC method. *CHII*, *CHI* leaves infusion; *CHIP*, *CHI* leaves powder; *CHIE*, *CHI* leaves extract. Data are presented as mean ± SEM (*n* = 2–3).

**TABLE 2:** Catechins content in *CHI* leaves infusion (1/10, m/v; mg/mL)^a^.

	Catechins						Total polyphenols
	**EGC**	**C**	**EC**	**EGCG**	**ECG**	**Total catechins**	
Contents	1.10 ± 0.04	0.65 ± 0.02	0.03 ± 0.00	0.03 ± 0.00	0.10 ± 0.00	1.91 ± 0.07	4.96 ± 0.04

^a^Total catechins represent the sum of epigallocatechin (EGC), catechin (C), epicatechin (EC), (-)-epigallocatechin-3-gallate (EGCG), and (-)-epicatechin-3-gallate (ECG). Catechins were measured with HPLC method. Total polyphenol was measured with folin-ciocalteu method. *CHII*, *CHI* leaves infusion. Data are presented as mean ± SEM (*n* = 3).

### *In vitro* antioxidant activity analysis

Total antioxidant activities of the *CHI* leaves infusion (*CHII*) were measured using commercial kits via FRAP and ABTS methods, following the methods adopted by Santos et al. (27).

### Animals and treatments

Kunming mice (18–20 g) were purchased from Shanghai SLAC Laboratory Animal Co., Ltd. (Shanghai, China). Golden hamsters (120–160 g) were purchased from Beijing Vital River Laboratory Animal Technology Co., Ltd. (Beijing, China). The regular chow diet (AIN-93) and high-fat diet (HFD) were provided by Trophic Animal Feed High-Tech Co., Ltd. (Nantong, China). All animals were housed in a room with a temperature of 24 ± 2°C, relative humidity of 50 ± 10%, and 12 h light/dark cycles, and free access to food and water *ad libitum*. All animal experimental protocols were reviewed and approved by the Animal Care and Ethics Committee of Anhui Agricultural University (ethics approval code, AAU 2018-054).

### *In vivo* experiments

#### Experiment 1

To evaluate the antioxidant capacity of *CHII in vivo*, the Kunming mice were divided into four groups (*n* = 6), allowed free access to water as control or *CHII* (1/40, *CHII*-L; 1/20, *CHII*-M; or 1/10, *CHII*-H; w/v) for 1 week, and then were sacrificed by cervical dislocation. Drinking fluids were refreshed daily.

#### Experiment 2

To evaluate the ameliorating effects of *CHII* on diabetic symptoms *in vivo*, diabetic mouse model was established through a single intraperitoneal injection of 200 mg/kg alloxan (ALX) to wild-type Kunming mice. One week after the ALX injection, mice with fasting blood glucose (FBG) at the range of 12–24 mmol/L were divided into three groups with even FBG levels, allowed free access to water as diabetic control or *CHII* (1/40, *CHII*-L; or 1/20, *CHII*-M; w/v). Wild-type Kunming mice were used as standard control (*n* = 6). Drinking fluids were refreshed daily. Mice were sacrificed by cervical dislocation after 2 weeks of treatment.

#### Experiment 3

To evaluate the long-term effects of *CHII* on diabetic symptoms, the diabetic mouse model was established through a single intraperitoneal injection of 200 mg/kg of ALX to wild-type Kunming mice. One week after the ALX injection, mice with FBG at 11–24 mmol/L were divided into two groups with even FBG levels, and allowed free access to water as diabetic control or *CHI* infusion (*CHII*-M, 1/20, w/v). Wild-type Kunming mice were used as standard control (*n* = 6). Drinking fluids were refreshed daily. Mice were sacrificed by cervical dislocation after 5 weeks.

#### Experiment 4

To evaluate the ameliorating effects of *CHIP* on hyperlipemia *in vivo*, a hyperlipemic model was established through HFD in golden hamsters. Golden hamsters were divided into four groups (*n* = 8), allowed free access to the regular chow diet as standard control, HFD, or *CHIP* (2% or 6% in HFD, m/m) for 9 weeks, and then were sacrificed by cervical dislocation.

#### Experiment 5

To evaluate ameliorating effects of *CHIE* on hyperlipemia and to evaluate regulating effects of *CHIE* on gut microbiota *in vivo*, golden hamsters were divided into four groups (*n* = 8); one group was allowed free access to the regular chow diet and water as standard control, other groups were allowed free access to HFD and water or *CHIE* (0.72% or 2.16% in drinking fluid, m/v) for 10 weeks, and then were sacrificed by cervical dislocation. Drinking fluids were refreshed daily.

### Sample preparation and biomarker assessments

The serum was centrifuged at 6,000 *g* at 4°C for 10 min. Serum ALT, AST, AKP, TC, TG, LDL-C, and HDL-C levels were measured using commercial kits. Fasting blood glucose levels of mice were measured after overnight fasting on tail vein blood with one touch glucometer (Roche Diagnostics, Mannheim, Germany). Hepatic tissues were excised, rinsed in ice-cold saline and in ice-cold phosphate buffer solution (PBS) at 0.15 mmol pH 7.2 containing 1 mmol ethylene diamine tetraacetic acid (EDTA), and then homogenized in ice-cold saline centrifuged at 15,000 *g* and 4°C for 15 min. Protein levels were determined by the Bradford dye-binding assay with BSA as the standard. GR activity was measured by the method of Carlberg and Mannervik with oxidized glutathione as a substrate (7, 28), and presented in terms of nmol of NADPH oxidized/min/mg protein. Glutathione S-transferase (GST) activity was assessed using CDNB and calculated as nmol CDNB conjugate formed/min/mg protein (29, 30). GPx and TrxR activities were determined using the Smith and Levander method with some modifications (31, 32). Glutathione peroxidase (GPx) activity was determined in working solution (65 mM PBS, pH 7.4 containing 2.5 mM GSH, 0.5 mM NADPH and 1.7 U/mL GR) and 3 μL hydrogen peroxide (H_2_O_2_), and was monitored by a microplate reader at 37°C and absorbance value of 340 nm. Thioredoxin reductase activity was determined in a working solution (200 mM PBS, pH 8.0 containing 2 mg/mL DTNB, 0.2 mg/mL NADPH and 0.2 mg/mL BSA), and was monitored by a microplate reader at 37°C and absorbance value of 412 nm. Glutathione peroxidase activity and TrxR activity were calculated in terms of μmol of NADPH oxidized/min/mL serum or mg protein.

### Western blot analysis

To extract total protein, livers or kidneys (0.1 g) were homogenized in 1 mL ice-cold RIPA buffer containing 0.1 mg/mL phenylmethylsulfonyl fluoride, and the lysates were clarified by centrifugation (15,000 *g*, 10 min, 4°C). To extract membrane protein, kidneys (0.1 g) were homogenized in 1 mL ice-cold membrane protein isolation buffer and then performed the guidelines provided by the manufacturer (Beyotime Biotechnology Co., Ltd., Shanghai, China). The total protein concentrations of supernatants were determined by the BCA protein assay kit. Equal amounts of protein were boiled at 95°C for 10 min in 5 × loading buffer, were separated through electrophoresis on a 10–15% sodium dodecyl sulfate-polyacrylamide gel electrophoresis (SDS-PAGE), and then were transferred to a PVDF membrane. After blocking with 5% skimmed milk in Tris-buffered saline with 0.05% Tween 20 (TBS-T) for 2 h at room temperature (RT), the membrane was incubated with specific primary antibody diluted in TBS-T by 200–5,000 folds overnight at 4°C, and then incubated with secondary antibody diluted in TBS-T by 5,000-fold for 1 h at RT after washing four times with TBS-T. The immunoreactivity was detected using the ChemiDoc XRS + detection system (ECL, Bio-Rad, USA) after being washed four times with TBS-T. The corresponding bands were quantified by densitometry with the Quantity One^®^ Image Analyzer software program (Bio-Rad).

### Gut microbiota profiling

The total genome DNA of bacteria was extracted with a fecal DNA isolation kit (MoBio Laboratories, USA) from frozen feces according to the manufacturer’s instructions. The 16S rDNA gene was amplified using a specific primer with the barcode (16S V3 + V4). DNA sequencing libraries were generated using an NEB Next Ultra DNA Library Prep Kit for Illumina (NEB, Ipswich, MA, USA). The PCR reaction conditions consisted of 95°C for 3 min (1 cycle), 95°C for 30 s and 50°C for 30 s as well as 72°C for 30 s (25 cycles), and a final extension at 72°C for 10 min in the presence of Fast Hifidelity Polymerase and Phusion^®^ High-Fidelity PCR Master Mix with GC Buffer (New England Biolabs Co., Ltd., Beijing, China). Paired-end sequencing of the PCR products was performed on a NovaSeq6000 at LC-Bio Technologies Co., Ltd. (Hangzhou, China).

### Statistical analysis

The data were expressed as mean ± standard error of the mean (SEM), and compared using one-way analysis of variance *post hoc* Tukey or Dunnett test as appropriate. All statistical analyses were performed using GraphPad software (Prism version 5, San Diego, CA, USA). The correlation coefficient of gut microbiota at the genus level was performed with Pearson correlation analysis (SPSS software, version 20, IBM, Armonk, NY, USA). A *p*-value lower than 0.05 was considered to be statistically significant.

## Results

### Flavonoids content and antioxidant activities of *CHII in vitro and in vivo*

**FIGURE 1:**
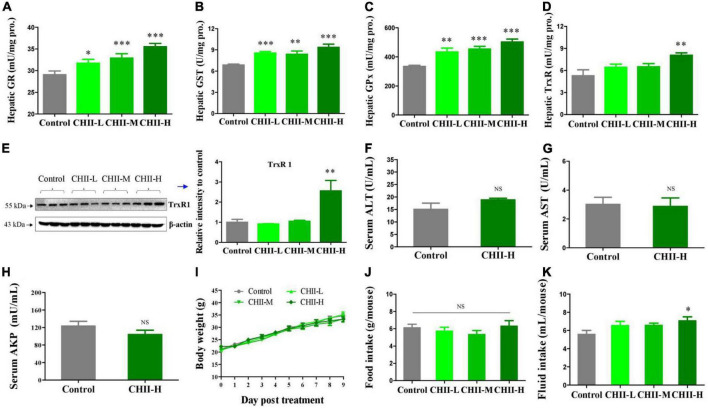
Antioxidant capacity and safety of *CHII* (1/10, 1/20, 1/40, m/v) in mice. Treatments were introduced in animal experiment 1. **(A–D)** Enzyme activity levels of GR, GST, GPx, and TrxR in liver. **(E)** TrxR1 protein level in liver. **(F–H)** Levels of ALT, AST, and AKP in serum. **(I–K)** Body weight and food and fluid intakes. *p < 0.05, **p < 0.01, ***p < 0.001; NS, *p* > 0.05, compared to control. Data are presented as mean ± SEM (*n* = 6).

The High-performance liquid chromatography (HPLC) profiling of three preparation samples demonstrated the presence of flavonoids in *CHI* leaves, which mainly include rutinum, isoquercitrin, kaempferol-3-O-rutinoside, astragalin, quercetin, and kaempferol (Table 1). In addition, catechins components were identified by HPLC in *CHII* sample (Table 2). The FRAP and ABTS assays were used to assay *CHII*’s total antioxidant activity *in vitro*. Results showed that *CHII* had a dose-dependent antioxidant property and an ABTS scavenging activity (Supplementary Figure 1). *In vivo, CHII* up-regulated the levels of the hepatic antioxidant enzyme activity, including GR, GST, and GPx (Figures 1A–C) in a dose-dependent manner (1/40, 1/20, and 1/10, m/v) in mice. Furthermore, the high-dose *CHII* (1/10, w/v) increased the levels of hepatic TrxR activity and TrxR1 protein expression (Figures 1D, E) without a significant effect on the levels of ALT, AST and AKP in serum, body weight, and food intake (Figures 1F–J), but increased fluid intake (Figure 1K). These results suggest that *CHII* can prevent oxidative stress-related diseases by strengthening the antioxidant defense system without increasing the risk of liver damage.

### *CHII* effectively ameliorated symptoms of diabetes in ALX-induced diabetic mice

**FIGURE 2:**
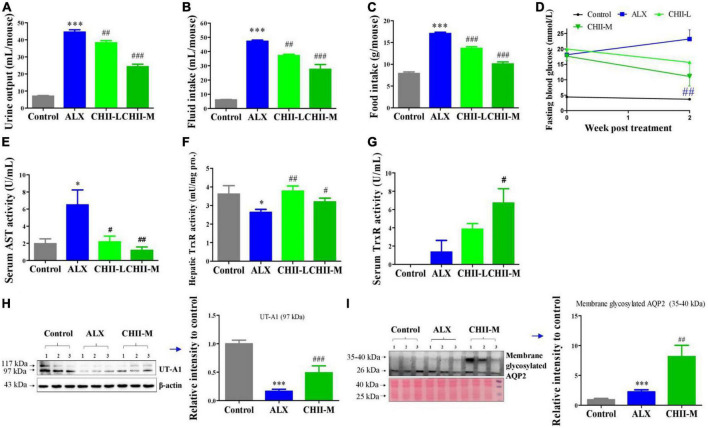
Effect and mechanism of short-term intake of *CHII* (1/20, 1/40, m/v) on symptoms of diabetes in ALX-induced type 1 diabetic mice. Treatments were introduced in animal experiment 2. **(A–C)** Urine output and fluid and food consumptions. **(D)** Fasting blood glucose. **(E)** AST in serum. **(F,G)** Enzyme activity levels of TrxR in liver and serum. **(H,I)** Protein levels of UT-A1 and membrane glycosylated AQP2 in kidney. *p < 0.05, ***p < 0.001, compared to control. ^#^*p* < 0.05, ^##^*p* < 0.01, ^###^*p* < 0.001, compared to ALX. NS, *p* > 0.05. Data are presented as mean ± SEM (*n* = 6–8).

Alloxan-induced type 1 diabetic mice were used to investigate the alleviating effects of *CHII* on typical diabetic symptoms. Results showed that *CHII* dose-dependently (1/40 and 1/20, m/v) improved the diabetic symptoms, including polyuria, polydipsia, polyphagia and hyperglycemia (Figures 2A–D), as indicated by the markedly reduced levels of urine output, fluid and food intakes and fasting blood glucose (Figures 2A–D) after treatment for 2 weeks. In addition, we found that alloxan treatment significantly elevated the level of AST in serum and suppressed the enzyme activity level of TrxR in the liver. Of interest, both doses of *CHII* decreased the level of AST in serum (Figure 2E) and increased the enzyme activity level of TrxR in the liver and serum (Figures 2F, G). At the molecular level, the ALX treatment significantly down-regulated renal water resorption-associated protein – UT-A1 (Figure 2H), which should induce diabetic polyuria and polydipsia. The medium-dose *CHII* prevented the down-regulation of renal UT-A1 (Figure 2H) and enhanced the tracking of AQP2 to the apical plasma membrane, as indicated by the increased expression level of membrane glycosylated AQP2 (Figure 2I). A long-term (5 weeks) experiment was conducted to confirm the ameliorating effects of *CHII* on diabetic symptoms and the regulating effect on renal water reabsorption associated proteins in ALX-induced diabetic mice.

**FIGURE 3:**
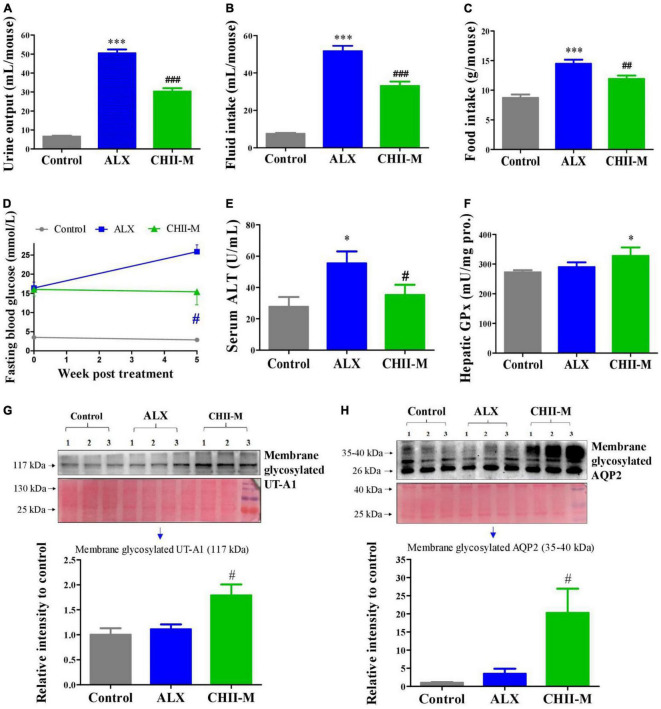
Effect and mechanism of long-term intake of *CHII* (1/20, m/v) on symptoms of diabetes in ALX-induced type 1 diabetic mice. Treatments were introduced in animal experiment 3. **(A–C)** Urine output and fluid and food consumptions. **(D)** Fasting blood glucose. **(E)** Level of ALT in serum. **(F)** Enzyme activity of GPx in liver. **(G,H)** Protein levels of membrane glycosylated UT-A1 and AQP2 in kidney. *p < 0.05, ***p < 0.001, compared to control. ^#^*p* < 0.05, ^##^*p* < 0.01, ^###^*p* < 0.001, compared to ALX. NS, *p* > 0.05. Data are presented as mean ± SEM (*n* = 6).

Again, results showed that *CHII* (1/20, m/v) pronouncedly ameliorated diabetic symptoms mentioned above (Figures 3A–D), prevented the elevation of ALT in serum (Figure 3E), enhanced the activity of GPx in the liver (Figure 3F), and significantly, increased the expression levels of renal membrane glycosylated UT-A1 and AQP2 (Figures 3G, H). Based on the present results, we conclude that *CHII* has a pronounced alleviating effect on diabetes. To further investigate the influence of different preparation forms of *CHI* leaves on metabolic syndrome, we explored the ameliorating effects of *CHI* leaves powder and *CHI* leaves extract on hyperlipidemia in golden hamsters fed a high-fat diet.

### *CHIP* did not pronouncedly alleviate hyperlipidemia in golden hamsters fed a high-fat diet

**FIGURE 4:**
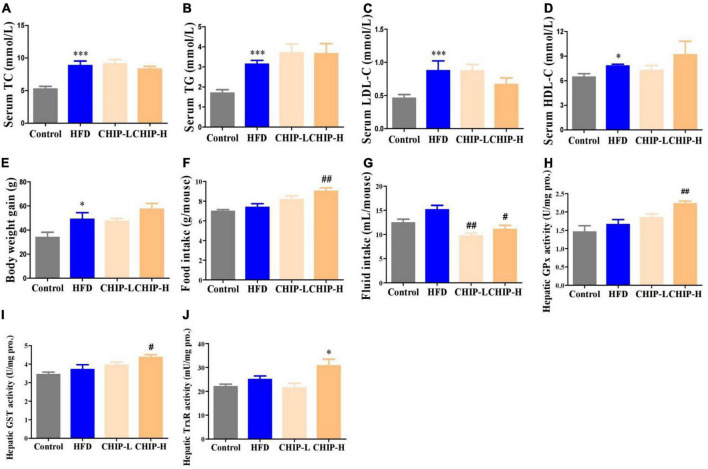
Effects of *CHIP* (2% or 6% in diet, m/m) on hyperlipidemia in golden hamsters fed a high-fat diet. Treatments were introduced in animal experiment 4. **(A–D)** Levels of TC, TG, LDL-C and HDL-C in serum. **(E–G)** Body weight gain and food and fluid intakes. **(H–J)** Enzyme activity levels of GPx, GST and TrxR in liver. *p < 0.05, ***p < 0.001, compared to control. ^#^*p* < 0.05, ^##^*p* < 0.01, compared to ALX. NS, *p* > 0.05. Data are presented as mean ± SEM (*n* = 4–8).

The high-fat diet elevated the levels of TC, TG, LDL-C, and HDL-C in serum (Figures 4A–D) and body weight gain (Figure 4E) without significantly altering the food and fluid intakes (Figures 4F, G) and the hepatic enzyme activity levels of GPx, GST and TrxR (Figures 4H–J) in golden hamsters. Surprisingly, *CHIP* (2% or 6% in diet, m/m) did not provide a significant effect on hyperlipidemia (Figures 4A–D) and body weight gain (Figure 4E), but the high-dose *CHIP* significantly enhanced the hepatic enzyme activity levels of GPx, GST and TrxR (Figures 4H–J). In addition, the high-dose *CHI* leaves powder increased food intake (Figure 4F), and both doses of *CHI* leaves powder significantly decreased the fluid intake (Figure 4G).

### *CHIE* markedly alleviated hyperlipidemia in golden hamsters fed a high-fat diet

**FIGURE 5:**
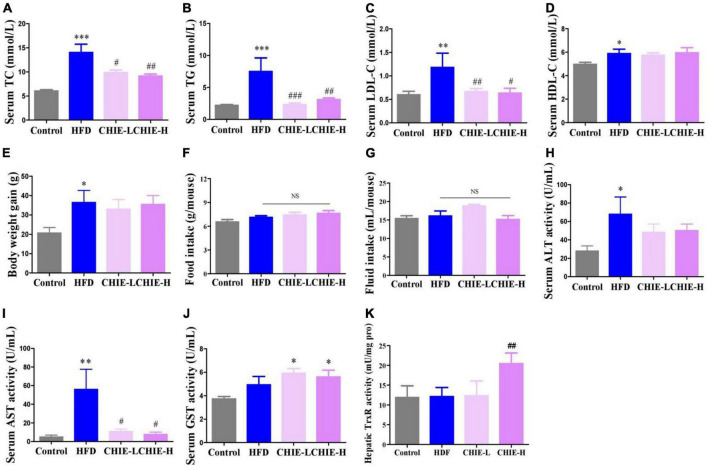
Effects of *CHIE* (0.72% or 2.16% in drinking fluid, m/v) on hyperlipidemia in golden hamsters fed a high-fat diet. Treatments were introduced in animal experiment 5. **(A–D)** Levels of TC, TG, LDL-C and HDL-C in serum. **(E–G)** Body weight gain and food and fluid intakes. **(H,I)** Levels of ALT and AST in serum. **(J,K)** Enzyme activity levels of GST and TrxR in liver. *p < 0.05, **p < 0.01, ***p < 0.001, compared to control. ^#^*p* < 0.05, ^##^*p* < 0.01, ^###^*p* < 0.001, compared to ALX. NS, *p* > 0.05. Data are presented as mean ± SEM (*n* = 8).

Interestingly, the elevated levels of TC, TG, and LDL-C (Figures 5A–C) induced by a high-fat diet were pronouncedly reduced (Figures 5A–C) by *CHIE* (0.72% in drinking fluid, m/v) that containing the lower dose of total flavonoid compared to the high-dose *CHIP* (6% in diet, m/m) (Table 1) without markedly changed the level of HDL-C in serum (Figure 5D), body weight gain (Figure 5E) and food and fluid intakes (Figures 5F, G) in golden hamsters. The markedly elevated levels of ALT and AST in serum (Figures 5H, I) indicated that a long-term high-fat diet increased the risk of liver damage. We found that both doses of *CHIE* (0.72%, 2.16% in drinking fluid, m/v) increased the enzyme activity level of GST (Figure 5J) and reduced the level of AST (Figure 5I) in serum. Furthermore, the high-dose *CHIE* increased the enzyme activity of TrxR (Figure 5K) in the liver.

### *CHIE* influenced gut microbiomes in golden hamsters fed a high-fat diet

**FIGURE 6:**
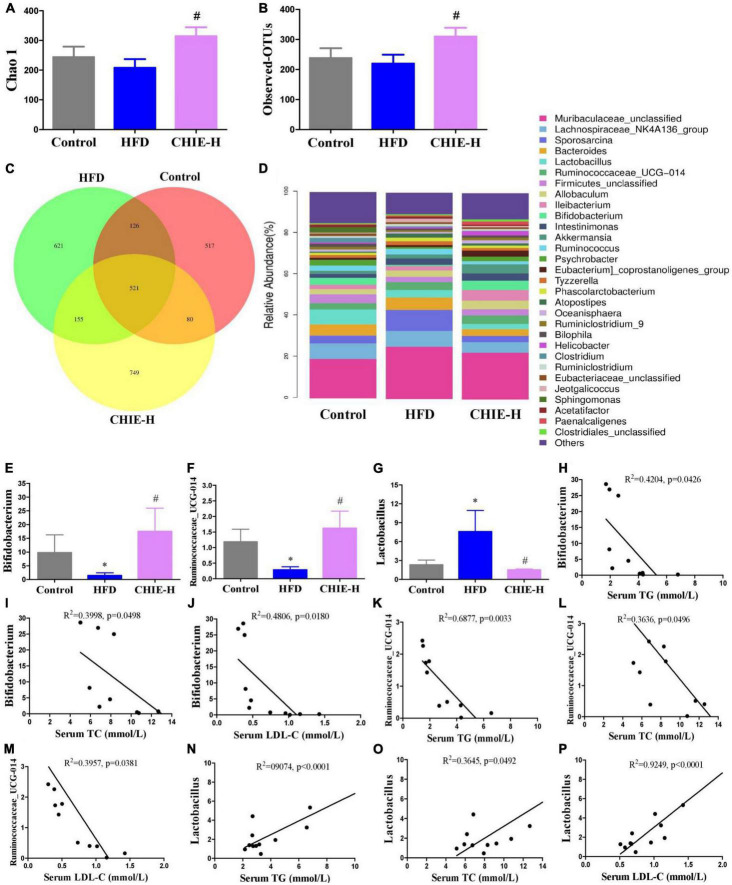
Effects of *CHIE* on gut microbiota in golden hamsters fed a high-fat diet and the correlation analyses between relative abundance of gut microbiota and hyperlipidemia core parameters. Treatments were introduced in animal experiment 5. **(A)** Chao1 index of alpha-diversity. **(B)** Observed OTU number. **(C)** Petal analysis of OTU. **(D)** Relative abundance of gut microbiota at the genus level (top 30). **(E–G)** Relative abundance of *Bifidobacterium*, *Ruminococcaceae_UCG-014* and *Lactobacillus* at the genus level in feces. **(H–P)** Correlation analyses between the relative abundance of *Bifidobacterium*, *Ruminococcaceae_UCG-014*, and *Lactobacillus* with the levels of TG, TC or/and LDL-C in serum. *p < 0.05, compared to control. ^#^*p* < 0.05, compared to HFD. NS, *p* > 0.05. Data are presented as mean ± SEM (*n* = 4).

In addition, we found that the high-dose *CHIE* pronouncedly influenced the community structure and relative abundance of gut microbiomes (Figures 6A–D). More specifically, the α-diversity analysis of the gut microbiomes showed *CHIE* increased microbiota diversity, evidenced by the elevated Chao1 index and observed species (Figures 6A, B). The similarity and consistency of samples were displayed with the overlapping operational taxonomic units (OTUs) in the Venn diagram. There are 521 OTUs shared in all groups. One thousand and 244 OTUs were identified in the control group, and a high-fat diet increased the OTUs numbers to 1,423. *CHIE* increased the OTUs numbers to 1,505 further (Figure 6C). In addition, *CHIE* increased the relative abundance of *Bifidobacterium* and *Ruminococcaceae_UCG-014*, and decreased the relative abundance of *Lactobacillus* in feces at the genus level in golden hamsters fed a high-fat diet (Figures 6E–G). Correlation analyses between the relative abundance of the markedly altered gut microbiota and hyperlipidemia-related parameters – TG, TC, and LDL-C – suggested that the abundances of *Bifidobacterium* and *Ruminococcaceae_UCG-014* are negatively (*p* < 0.05) correlated with the levels of TG, TC and LDL-C in serum (Figures 6H–M), and the abundance of *Lactobacillus* is positively (*p* < 0.05) correlated with the levels of TG, TC and LDL-C in serum (Figures 6N–P).

## Discussion

Trx and GSH systems are the two well-documented NADPH-dependent disulfide reduction pathways and serve as backup systems for each other (33). Nrf2 pathway is known as a regulator of redox homeostasis, whereas, TrxR1, Trx1, GST, and GPx, as the critical components of Trx and GSH systems, are Nrf2 target genes (7, 34, 35). In addition, the activation of heme oxygenase 1 (HO-1) and NAD(P)H: quinone oxidoreductase 1 (NQO1), the downstream genes of Nrf2, depend on the activity of TrxR1, which has been proved with direct inhibition with TrxR1 siRNA and chemical inhibitor studies (36, 37). Nrf2 activation via TrxR1 suppression represents a strategy shift for oxidative injury treatment (38). Overall, there is an intricate interaction among antioxidant defense systems. A variety of polyphenols could induce the antioxidant defense systems mentioned above. For instance, rutinum, astragalin, and quercetin can up-regulate the Nrf2 pathway, as evidenced by the augmented activity of GPx, GR, and GST (39–41) and detoxification enzyme HO-1 and NQO1 (42, 43). Isoquercitrin can activate Nrf2 pathway as reflected by the induction of HO-1 and NQO1 (44, 45). Catechins, including EGC, C, and EGCG, possess the capacity of Nrf2 and its target genes activation, including TrxR1 and GPx1 (7, 46, 47). Our data showed *CHI* leaves increased the levels of GR, GST, GPx, and TrxR enzymes activity as well as TrxR1 protein in the liver (Figures 1A–E) in healthy mice, TrxR and GPx enzymes activity in the liver and TrxR activity in serum (Figures 2F, G, 3F) in ALX-induced type 1 diabetic mice; and GPx, GST and TrxR enzymes activity in the liver (Figures 4H–J, 5K), as well as GST activity in serum (Figure 5J) in golden hamsters, fed a high-fat diet. These results suggest that the activation of Trx and GSH systems may be attributed to the high amount of flavonoids and catechins in *CHI* leaves (Tables 1, 2), and the activation of Trx and GSH systems may contribute to the up-regulation of the upstream-Nrf2.

Numerous reports showed that the induction of antioxidant defense systems is beneficial to oxidative damage and inflammation response prevention, thereby, alleviating metabolic syndrome (48–50). Besides, Nrf2 inhibits adipogenic differentiation via activation of the aryl hydrocarbon receptor (AHR) pathway or stimulation of GSH metabolism (51), suggesting that the enhancement of antioxidant defense systems may contribute to ameliorating lipid metabolic abnormality. Indeed, in the present study, we found *CHI* had a protective effect on liver injury, as reflected by the reduced levels of AST in serum (Figures 2E, F, 3E, 5H–I) in murine with glucose or/and lipid dysmetabolism induced by ALX or high-fat diet. In addition, *CHI* markedly alleviated hyperglycemia, as evidenced by the decreased fasting blood glucose in ALX-induced type 1 diabetic mice (Figures 2D, 3D), and hyperlipidemia, as reflected by the reduced levels of TC, TG, and LDL-C (Figures 5A–C) in serum in golden hamsters fed a high-fat diet, which suggests *CHI* is beneficial to glycolipid dysmetabolism alleviation *in vivo*. Nevertheless, the causal relationship between oxidative stress reduction and glycolipid dysmetabolism improvement needs further investigation.

Diabetes mellitus is a debilitating disease with multiple symptoms, including hyperglycemia, polyuria, polydipsia, and polyphagia in the clinic. Polyuria and glycosuria as the first symptoms trigger polydipsia and hyperphagia to maintain water and energy balance. However, hyperphagia could further elevate blood glucose which induces osmotic diuresis leading to a vicious cycle. Ultimately, these extremely exhausted pathophysiological responses fail to alleviate advanced glycemia. Metformin, as the first line and the most commonly prescribed drug, possesses significant advantages in hypoglycemic efficacy, high safety and low cost for the therapy of T2DM (52–55). However, metformin exhibits a weak diabetic polydipsia alleviation effect (56, 57). Rosiglitazone, another anti-diabetes oral drug, can cause serious fluid retention, plasma volume expansion, and large body weight gain in humans and db/db mice (58–61) though pronouncedly alleviated the polydipsia in diabetic animals (58–61). Overall, there are various side effects in currently available anti-diabetic drugs, leading to an incongruously low proportion of patients achieving glycemic goals with single or multiple anti-diabetic agents. Therefore, it is of great significance to search for natural products with glycolipid regulatory ability from natural products pool being enriched with bioactive compounds and acting as a crucial role in drug discovery for the prevention or improvement of metabolic syndrome. In this study, we found *CHI* leaves improved diabetic symptoms, including polyuria, polydipsia, polyphagia, and hyperglycemia, as reflected by the decreased levels of urine output, fluid and food intake as well as fasting blood glucose (Figures 2A–D, 3A–D) after treatment for 2 or 5 weeks. These results suggest that *CHI* leaves can ameliorate typical symptoms of diabetes, especially polyuria and polydipsia, rapidly and persistently without observable side effects.

The kidney maintains glucose and body fluid homeostasis, and reabsorbs 99% of raw urine (about 180 L) produced by healthy adults per day. Renal water reabsorption-associated proteins, including UT-A1 and AQP2, regulated by PKC-α via phosphorylation of UT-A1 at Ser494 and AQP2 at Ser256 or dephosphorylation of AQP2 at Ser261 (62–67), in medullary collecting ducts play an important role in maintaining body water homeostasis via promoting renal water reabsorption and urine concentration (68, 69). For instance, lack of urea transporter UT-B adaptively increased the expression of AQP2 in UT-B null mice (69). AQP2 and UT-A1 proteins were up-regulated in the renal medulla collecting duct in diabetes for fighting against urine-concentrating defect (68, 70). And the down-regulation of AQP2 and UT-A1 pronouncedly elevated the urine output in lithium-induced nephrogenic diabetes insipidus (71). We hypothesized that it is a crucial strategy for blocking glucose-induced osmotic diuresis by activating renal water reabsorption-associated proteins in diabetes with symptoms of polyuria and polydipsia. Indeed, in this work, we found *CHI* leaves could quickly and markedly alleviate diabetic polyuria and polydipsia in a dose-dependent manner within 2 weeks post-treatment in diabetic mice (Figures 2A, B), and the mechanism involved in the enhanced renal water reabsorption associated protein UT-A1 and the increased trafficking of AQP2 to the apical plasma membrane (Figures 2H, I). These bioeffects above mentioned and the responses of renal UT-A1 and AQP2 to *CHI* leaves consumption were also observed in the long-term experiment in diabetic mice (Figures 3A–D, G, H). The unique biological activity of *CHI* leaves on up-regulating renal water reabsorption-associated proteins suggests that *CHI* leaves may act as a dietary supplement for ameliorating diabetic polyuria and polydipsia.

Several reports showed that polyphenols, including green tea polyphenol preparation and epicatechin, reduced the fluid intake in diabetic mice (72, 73). However, the active ingredients activate renal water reabsorption-associated proteins are still unclear, because these reports mentioned above related their results to the reduced fasting blood glucose and glucose-induced osmotic diuresis. Until recently we demonstrated that EGCG pronouncedly up-regulated renal water reabsorption-associated proteins involved in urine concentration and reduced urine output (74), which suggested EGCG can block glucose-induced osmotic diuresis via up-regulating renal water reabsorption associated proteins because urine output is positively correlated with fluid intake (74). Since a limited amount of EGCG in *CHI* leaves infusion (0.03 mg/mL, 1/10, m/v) (Table 2), certain phytochemicals other than EGCG in *CHI* leaves infusion are involved in the up-regulation of renal water reabsorption associated proteins. This requires further investigation and the evidence mentioned above suggests the water-soluble polyphenols in *CHI* leaves infusion should be an essential clue.

*Bifidobacterium* and *Ruminococcaceae_UCG-014* are known as probiotics. *Bifidobacterium* participates in the alleviation of oxidative stress (75) and the production of short-chain fatty acids (SCFAs) in the gut (76), and shows glycosidic activity in enhancing the bioavailability of isoflavone *in vivo* (77, 78). *Ruminococcaceae_UCG-014* is beneficial SCFAs-producing bacteria (79), which was reported to affect relieving high-fat diet-induced obesity (80), type 2 diabetes in rats (81) and dextran sodium sulfate induced homeostasis imbalance of host health in mice (82). We found high-fat diet markedly decreased the relative abundance of *Bifidobacterium* and *Ruminococcaceae_UCG-014* at the genus level in feces, but *CHI* leaves extract effectively prevented the reduction of these probiotics (Figures 6E, F). *Lactobacillus* is beneficial bacteria for promoting digestion, maintaining intestinal flora homeostasis and preventing metabolic syndrome (83, 84). Interestingly, reports show high-fat diet or nutrient overload increases the abundance of *Lactobacillus* in the gut (85, 86), which might be attributed to the fact that excessive calory is beneficial to the proliferation of *Lactobacillus*. In this study, we found high-fat diet pronouncedly increased the relative abundance of *Lactobacillus* at the genus level; *CHI* leaves extract prevented the alteration (Figure 6G). In addition, correlation analyses showed that the abundances of *Bifidobacterium* and *Ruminococcaceae_UCG-014* are negatively correlated with the levels of TG, TC and LDL-C in serum (Figures 6H–M), and the abundance of *Lactobacillus* is positively correlated with the levels of TG, TC and LDL-C in serum (Figures 6N–P), which suggested *CHIE* reprogrammed gut microbiota contributed to the alleviation of hyperlipidemia that induced by a high-fat diet.

Food intake affects energy homeostasis seriously and calorie consumption is one of the significant determinants for metabolic outcome. In this study, we found *CHII* blocked glycosuria-caused calorie loss and compensatory polyphagia (Figures 2A, C, 3A, C) via promoting urine concentration (Figures 2A, 3A) by up-regulation of renal water reabsorption associated proteins (Figures 2H, I, 3G, H), thereby, as a consequence, hyperglycemia was alleviated pronouncedly (Figures 2D, 3D) in alloxan-induced diabetic mice. *CHIP* did not help hyperlipidemia in golden hamsters fed a high-fat diet (Figures 4A–C), which could be attributed to the increased food intake (Figure 4F). However, *CHIE* effectively improved hyperlipidemia (Figures 5A–C) by modified gut microbiota (Figures 6A–G) though *CHIE* did not significantly alter the calories intake in golden hamsters fed a high-fat diet (Figure 5F). This suggests that food consumption and gut microbiota are essential factors that should be considered when analyzing and interpreting the bioeffects and outcome of metabolic syndrome. Since different preparation forms may produce other biological effects (87), the present results suggested that *CHIE* may be a better form than *CHIP* for alleviating hyperlipidemia. Besides, in the present study, *CHII*, *CHIE*, and *CHIP* designed separate experiments, but proved the same hypothesis, which may not rigorous enough. Using three samples in one experiment to illustrate the same question will be more convincing. We stated this as the limitation of our experimental design.

## Conclusion

**FIGURE 7:**
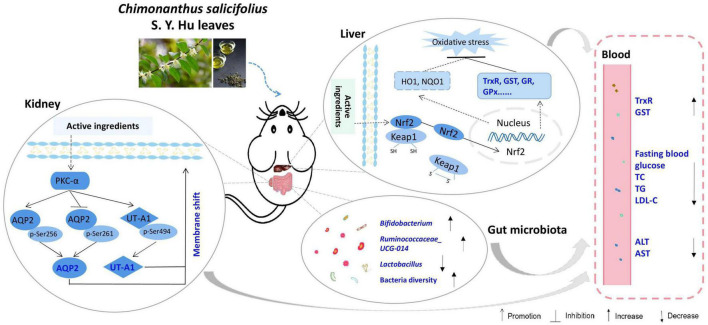
Schematic diagram showing the underlying mechanisms of *CHI* leaves consumption on ameliorating metabolic syndrome.

In summary, *CHII* showed strong antioxidant activity; as evidenced by the total antioxidant and free radical scavenging capacities measured by the FRAP and ABTS methods *in vitro* and the activated antioxidant defense systems–Trx and GSH systems–in liver and serum post *CHII* consumption in mice. *CHII* effectively alleviated diabetic symptoms, including polyurine, polydipsia, polyphagia and hyperglycemia, by up-regulating renal water reabsorption associated protein involved in urine concentration–AQP2 and UT-A1–and promoting tracking of these proteins to the apical plasma membrane in alloxan-induced diabetic mice. *CHIE* significantly reduced the levels of TC, TG, and LDL-C in serum by increasing the diversity of intestinal flora and the abundance of probiotics in the gut in golden hamsters fed a high-fat diet. Taken together, *CHI* leaves can effectively prevent oxidative stress by activating the antioxidant defense systems, improve diabetes by up-regulation of renal water reabsorption proteins and ameliorate hyperlipidemia via modifying gut microbiota (Figure 7), which suggests *CHI* leaves or their extract may be as prebiotics for preventing overnutrition-associated disease, including oxidative stress and metabolic syndrome, if these bioeffects mentioned above could be observed in humans.

## Data availability statement

The datasets presented in this study can be found in online repositories. The names of the repository/repositories and accession number can be found below: https://www.ncbi.nlm.nih.gov/sra/PRJNA953761.

## Ethics statement

All animal experimental protocols were reviewed and approved by the Animal Care and Ethics Committee of Anhui Agricultural University (ethics approval code, AAU 2018-054).

## Author contributions

RD and GZ: conceptualization, validation, supervision, and project administration. GZ, RD, JP, SW, SM, NL, LS, XH, JY, QZ, and FW: methodology. RD, JP, SW, LS, FW, and DW: investigation. GZ, RD, and QB: writing—original draft preparation and funding acquisition. GZ, DG, DW, RD, KC, and QB: writing—review and editing. All authors contributed to the article and approved the submitted version.
