# Supplementary material for: Antioxidant, antihyperglycemic, and antihyperlipidemic properties of Chimonanthus salicifolius S. Y. Hu leaves in experimental animals: modulation of thioredoxin and glutathione systems, renal water reabsorption, and gut microbiota
Source: Front Nutr. 2023 Apr 28;10:1168049. doi: 10.3389/fnut.2023.1168049 (PMC10176510; doi:10.3389/fnut.2023.1168049)
Supplement: Supplementary file 1 [file Data_Sheet_1.doc]

**Antioxidant, antihyperglycemic, and antihyperlipidemic properties of *Chimonanthus salicifolius* S. Y. Hu leaves in experimental animals: modulation of thioredoxin and glutathione systems, renal water reabsorption, and gut microbiota**

Ruixia Dong^1,2,3^†, Junjie Pan^4^†, Guangshan Zhao^3,5^*,Qiuyan Zhao^5^, Shiqiong Wang^5^, Ning Li^5^, Lianjun Song^5^, Xianqing Huang^5^, Shuxing Miao^1^, Junhui Ying^2^, Fangying Wu^2^,Dongxu Wang^3,6^*,Kejun Cheng^4^*, Daniel Granato^7^* and Qiuyan Ban^8^*

^1^College of Horticulture, Jinling Institute of Technology, Nanjing, China

^2^College of Forestry Science and Technology, Lishui Vocational and Technical College, Lishui, China

^3^State Key Laboratory of TeaPlant Biology and Utilization, School of Tea and Food Science and Technology, Anhui Agricultural University, Hefei, China

^4^Chemical Biology Center, Lishui Institute of Agriculture and Forestry Sciences, Lishui, China

^5^ Innovation Team of Food Nutrition and Safety Control, College of Food Science and

Technology, Henan Agricultural University, Zhengzhou, China

^6^School of Grain Science and Technology, Jiangsu University of Science and Technology, Zhenjiang, China

^7^Bioactivity and Applications Lab, Department of Biological Sciences, Faculty of Science and Engineering, University of Limerick, Limerick, Ireland

^8^Department of Tea Science, College of Horticulture, Henan Agricultural University, Zhengzhou, China

# These authors contributed equally to this work.

Corresponding authors: Guangshan Zhao (E-mail: zgs2015@yeah.net), Dongxu Wang (E-mail: wdx@just.edu.cn), Kejun Cheng (E-mail: chengkejun@gmail.com), Daniel Granato (E-mail: daniel.granato@ul.ie), and Qiuyan Ban (E-mail: banqiuyan717@163.com).


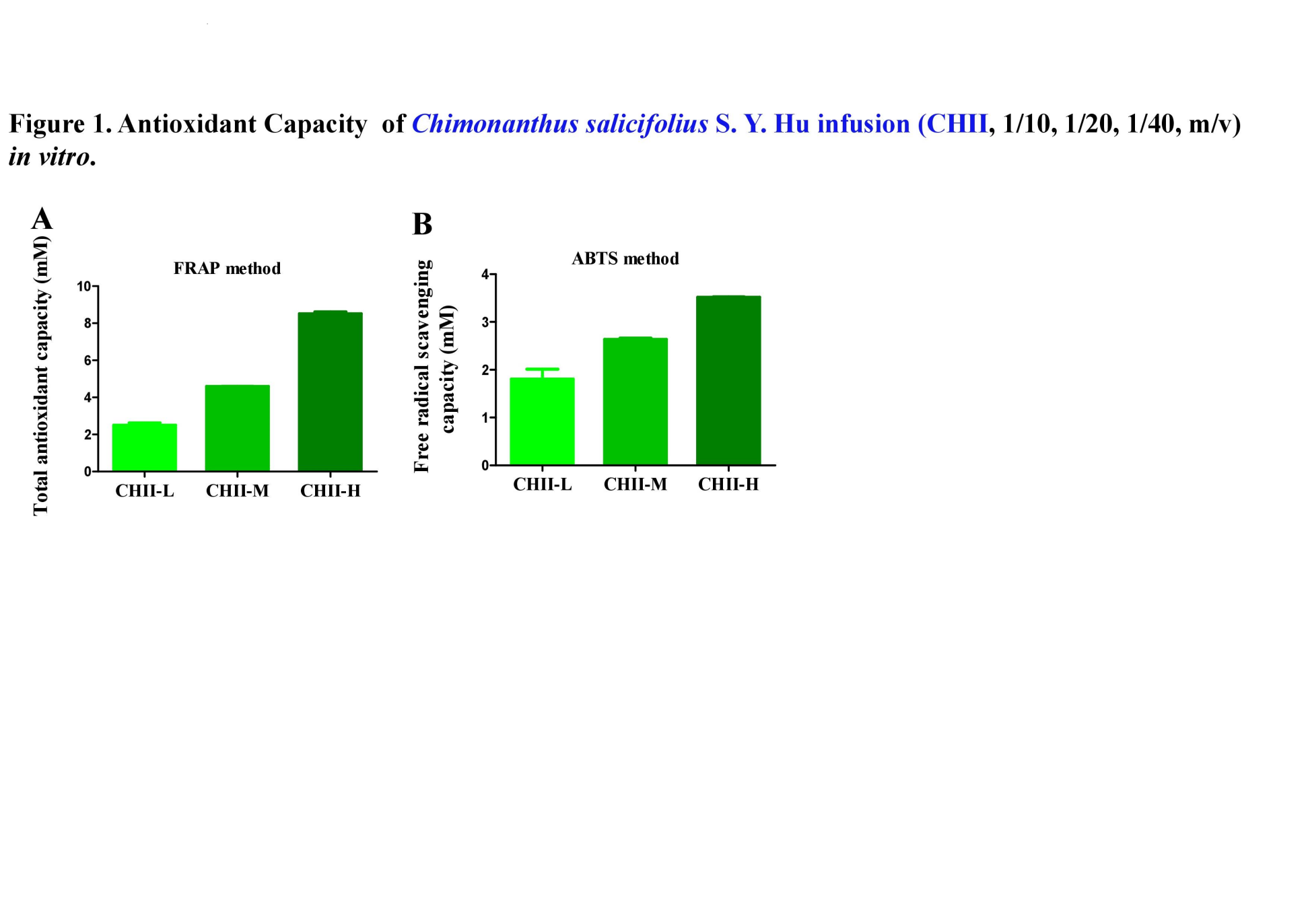


**Supplementary Figure 1. Total antioxidant and free radical scavenging capacities of *CHII* (1/10, *CHII*-H; 1/20, *CHII*-M; 1/40, *CHII*-L; m/v) *in vitro***. Total antioxidant (A) and free radical scavenging (B) capacities. Data are presented as mean ± SEM (n=3).
